# Infection, Celestial Influences, and Sudden Infant Death Syndrome: A New Paradigm

**DOI:** 10.7759/cureus.17449

**Published:** 2021-08-26

**Authors:** Paul N Goldwater, Edward O Oberg

**Affiliations:** 1 Pathology-Infectious Diseases and Clinical Microbiology, Adelaide Medical School, University of Adelaide, Adelaide, AUS; 2 Mechanical Engineering, University of Minnesota, Minnesota, USA

**Keywords:** sudden infant death syndrome, sids, celestial, infection, heliobiology

## Abstract

The etiology of sudden infant death syndrome (SIDS) still remains unclear. This situation would seem unprecedented for 21st-century medical science. This article explores scientific fields that have not been largely considered in investigating the etiology of SIDS so far. In this study, we examined previously ignored studies on heliobiology, celestial influences, and SIDS in the non-medical literature in an attempt to answer the following questions: is there a relationship between sunspot/solar activity and the occurrence of SIDS? Could there be alternative reasons for the decline in SIDS incidences in the 1990s that were originally attributed to the “Back-to-Sleep” campaign? We note that the decline coincided with the ~11-year cyclical diminution in sunspot numbers (SSNs). The SSN/SIDS relationship does not necessarily imply causality; however, it supports published data regarding sunspots, Schumann resonance, and geomagnetic effects. How solar energy could adversely influence a baby’s existence remains conjectural. Observations in this respect suggest pathways involving melatonin and/or infection/inflammation.

## Introduction and background

There are a number of definitions of sudden infant death syndrome (SIDS), which were coined in 1969, 1989, and 2004. These are known as the Seattle, National Institute of Child Health and Development (NICHD), and San Diego definitions, respectively, and are as follows:

Seattle (Beckwith) definition (1969): “the sudden death of any infant or young child which is unexpected by history, and in which a thorough post-mortem examination fails to demonstrate an adequate cause of death” [1].

NICHD (Willinger) definition (1989): “the sudden death of an infant under one year of age, which remains unexplained after a thorough case investigation, including the performance of a complete autopsy, examination of the death scene, and a review of the clinical history” [2].

San Diego definition (2004): “the sudden and unexpected death of an infant under one year of age, with the onset of the lethal episode, apparently occurring during sleep, that remains unexplained after a thorough investigation, including a performance of a complete autopsy, and review of the circumstances of death and the clinical history” [3].

The various definitions of SIDS are broadly similar in nature. They are definitions of exclusion and are thus unhelpful in providing pointers to the underlying cause or causes and, consequently, in devising appropriate approaches to address the problem. The San Diego definition has subcategories that are more helpful in that these account for the pathological aspects of the case in question. The emphasis on the “lethal episode, apparently occurring during sleep” may have been a disadvantage to researchers as it concentrated on physiological events occurring during an infant’s sleep rather than carefully dissecting the gross pathological and clinicophysiological findings and SIDS epidemiology, which could have provided direct clues to the underlying lethal process. This process is suggestive of infection/inflammation. Several factors contribute to the development of infection. This study examines a neglected one: the celestial phenomenon. Before examining this, it is important to understand the fundamental facts about SIDS. Primarily, it is critical to have an understanding of the pathology of SIDS (this has been wrongly assumed to be unremarkable by mainstream SIDS researchers).

The pathological findings [4] include the following: heavy fluid-laden lungs with early subtle acute inflammatory changes; intrathoracic petechial hemorrhages in and on the thymus, epicardium, and visceral pleura/lungs; liquid blood in the chambers of the heart; heavier than normal thymus [5,6], brain [5,7-14], and liver [5,14,15]; empty bladder; and raised core temperature [16].

Of equal importance are the clinicophysiological findings [17] obtained by using computer memory monitors attached to babies who died (perversely “serendipitously”) of a definitional SIDS event. These recordings have definitively shown that bradycardia was followed by asystole. Moreover, these cardiological events occurred before the commencement of gasping respirations and cessation of breathing. This suggests that the primary problem involves the heart, rather than the lungs.

In addition, there are noteworthy laboratory findings that contribute to the development of a sensible research pathway: raised serum fibrin degradation products (FDPs) [18], lower normal serum melatonin [19], and increased tissue inflammatory cytokines interferon-alpha, tumor necrosis factor, and interleukin 6 (IL-6) [20-24], including increased IL-6 in the cerebrospinal fluid [21] and eye vitreous [25].

Additional avenues remain to be investigated. These include the fact that infection and lethal sepsis stimulate the release of serotonin, resulting in increased serum levels [26], and serotonin has been the focus of intense research but without any apposite or conclusive results [27]. Blood serotonin levels have denoted conflicting results. Studies show that these are raised in cases of SIDS [28], while [A8] levels of tryptophan hydroxylase in the brainstem and serotonin receptor binding were lowered, which seems counterintuitive [27]; however, no important correlations with SIDS risk factors could be made.

In an attempt to fashion a research direction, a number of models have been proposed to incorporate the known SIDS risk factors [29-31]. These models resulted in the idea of the “triple risk” model of SIDS [29,32], which supposes that the risk of SIDS is increased when a vulnerable infant is exposed to environmental stressors. The three components of the model are as follows: (1) a critical developmental period (especially the first two to four months, the “SIDS peak”); (2) exposure to stressors (overheating, infection); and (3) underlying susceptibilities (age, sex, race, etc.) [32]. This model has since been refined, but its essence remains much the same [33].

The discovery of abnormalities in the brainstems of 40-50% of cases of SIDS resulted in further investigations into a common mechanism that could explain an alleged failure in homeostatic control (breathing and/or cardiac arrhythmia). The direction taken by mainstream SIDS researchers has focused on homeostatic control and has largely ignored the key epidemiological and clinicopathological findings enumerated above. Given the physiological monitoring findings related to cardiac control, such investigations are urgently needed, but the evidence is lacking for the argument that abnormal respiratory control is a primary lethal event. Evidence of chronic hypoxia in a proportion of SIDS cases [4,34] may have misled researchers into adopting a respiratory-based paradigm, but the evidence pertaining to “chronic hypoxia” is often contradictory [35]. Indeed, it seems that infection is correlated with increased vitreous hypoxanthine levels, and thus an infective mechanism could explain this phenomenon [25].

The sleeping position of babies has been featured in much of recent and current SIDS research. Despite the lack of direct evidence, the prone sleep position has been posited as having a causal relationship with mortality [36]. Such uncorroborated statements require serious dismantling (see below).

The clinicopathological findings suggest the following. Firstly, none are incompatible with infection, either acting as an instigating event or as a contributor to the lethal event. The supporting evidence for the “infection paradigm” includes male sex, prematurity, lack of breastfeeding, seasonality, waning maternal transplacental immunoglobulin G (IgG), exposure to contaminated surfaces/personal contact (used mattresses, co-sleeping in parental bed, sofa sleeping), overcrowding or low socioeconomic status, high birth order (older siblings expose the infant to respiratory viral infection), and prone sleep position (the effect of this appears only to operate when there is a coincidental infection) [37-39].

The important role of infection in SIDS has been described previously [40] and cannot be overstated [41]. A plausible mechanism would be an abnormal response to viral respiratory infection when the infant is challenged by a bacterial toxin. Experimental evidence was neatly demonstrated by the Nobel Laureate Peter Doherty and his colleagues in an experiment where mice were exposed to a virus and challenged with a staphylococcal enterotoxin. The mice died of hematogenous shock due to the dual exposure but did not die when exposed to the single agents [42].

Despite some claims to the contrary, the current overwhelming consensus is that the causes of SIDS remain undefined. The common hypotheses of mainstream researchers focus on the theory of homeostatic control of respiration and cardiac function. However, this approach has yet to provide a definitive explanation. Unlike the infection model, brainstem and other brain site data generally fail to link to known epidemiological SIDS risk factors, and even when links are made, these tend to be piecemeal and often contradictory [40]. For example, mainstream researchers failed to acknowledge the previously well-documented association with a respiratory viral infection. Also overlooked was the key epidemiological finding in relation to the prone sleep position demonstrated by the Tasmanian SIDS study [37]. This study revealed that the risk of SIDS associated with the prone sleep position increased 10-fold when there was a concurrent infection in the infant [36]. A Nordic study showed a 29-fold increase in risk due to prone plus infection [38,39]. Moreover, the infection paradigm is supported by and is congruent with all epidemiological SIDS risk factors [40,41]. It remains unclear as to why the infection hypothesis has not achieved greater prominence.

While it is clear that infection plays a role in the SIDS lethal pathway, it has been almost impossible to definitively associate a diagnosis of SIDS or sudden unexplained infant death (SUID) with infection per se, such as by establishing a diagnosis of early neonatal sepsis, as cultures are often negative [43]. However, the absence of findings does not necessarily mean the absence of infection. Should the search be extended further afield to find an explanation? Could there be external influences that combine with infection to tip the balance between life and death?

## Review

Celestial influences

This is the point at which consideration of celestial influences such as sunspots and other electromagnetic activity becomes apposite. In addition to the evidence laid out here and elsewhere, it has been known since the 1970s that the epidemic curve of influenza A closely follows that of SIDS [44], and this is supported by serological evidence [45]; influenza A epidemics appear to be associated with increased sunspot activity [46], but this association has been undermined on statistical grounds [47]. If we ignore the perhaps discredited epidemiological link between influenza A and sunspots, serological evidence [45] certainly provides a basis to explore a possible linkage between SIDS and sunspots/solar activity and, possibly, other celestial phenomena. An extensive literature search revealed several papers in the geophysical realm that have shown an association between SIDS and sunspots and related geomagnetic activity [48-52].

These studies have been largely overlooked by medical science. These reports indicate that celestial activity, particularly solar activity, could have direct or indirect lethal effects on susceptible human infants. Support for this idea is seen in sunspot-related celestial phenomena with meteorological, geological (earthquakes), and biological effects (sometimes lethal) [53-57].

The quest to establish a widely accepted cause for SIDS has been long and elusive. In this regard, in many ways, it is surprising that the papers of Cherry [51,53], O’Connor and Persinger [48-50], and Dupont et al. [52] have not been acknowledged by or not sparked the interest of mainstream SIDS researchers. However, approximately a decade before the work of O’Connor and Persinger on SIDS and celestial effects, Eckert [58] put forward a hypothesis that caught the interest of mainstream SIDS researchers, based upon observed clustering of SIDS cases at places with abnormal geomagnetic fields (GMF) and/or electromagnetic fields (EMF), and recordings of GMF with pulsations matching the breathing frequencies of infants. The reported immature development of increased dendritic spine density in the brain stem of SIDS cases and the increased dendrite arborization in the brains of rats exposed to magnetic fields also formed part of the hypothesis [59]. The hypothesis was tested by Grainger et al. [60], who conducted a small case-control study measuring 50 Hz electric and magnetic fields at the SIDS babies' last head position. No association was found between SIDS and either electric (p=0.327) or magnetic (p=0.827) 50 Hz fields. The study has received considerable criticism given the small case numbers, the restricted frequency band chosen for the study, and the lack of data on the compass orientation of the head or body of each case, and of actual geophysical measurements. Thereafter, mainstream SIDS research on electric and magnetic fields appeared to have ceased.

**Figure 1:**
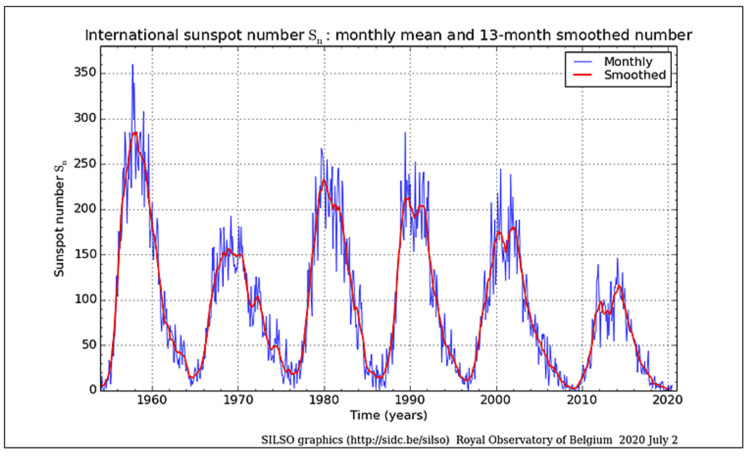
Monthly international sunspot numbers, 1960-2019

The decline in SIDS numbers in the 1990s has been attributed to the “Back-to-Sleep” campaign [61,62]. However, the decline also coincides with the ~11-year cyclical diminution in sunspot numbers (SSNs), which could contribute to or suggest evidence of a direct relationship to the SIDS decline (Figure 1). While this relationship does not necessarily imply causality, such a finding supports the previously published data regarding sunspots [48,51,53] and by inference to Schumann resonance [51,53] and geomagnetic effects [48-50,52], which are manifestations of solar electromagnetic activity.

According to the National Aeronautics and Space Administration (NASA), approximately 2,000 thunderstorms occur worldwide at any given moment. These storms produce approximately 50 flashes of lightning every second. Each lightning burst induces electromagnetic waves that encircle the Earth. These extremely low frequency (ELF) waves are captured between the Earth’s surface and the boundary of the ionosphere ~100 km above the surface. Some of the waves with the appropriate wavelengths combine and increase in strength to create a repeating heartbeat known as the Schumann resonance [63].

How this solar energy could adversely influence a human baby’s existence remains a subject of debate. One observation suggests that the pathways could involve melatonin [18], especially as the majority of SIDS cases occur at night, and these infants appear to have abnormally low melatonin levels [64].

Visible light switches off melatonin synthesis by the pineal gland [65], but the synthesis of melatonin can also be diminished by exposure to ELF electric and magnetic fields [66]. Considering that the majority of SIDS cases occur at night, it is reasonable to assume that exposure to light prior to death was not responsible for diminished serum levels of melatonin in these babies. This leaves us to speculate that other mechanisms are involved, possibly ELF EMFs. Studies have revealed that low-level, ELF electric and magnetic fields also affect circadian melatonin production [67]. Along with visible light, life on the Earth has been exposed to geomagnetic and geoelectric fields for hundreds of millions of years. These fields have strengths of 0.2-0.7 G, and approximately 100 volts/meter (V/m), respectively, at the Earth’s surface under good weather conditions and approaching several thousands of V/m under thunderstorm conditions. Wilson et al. (1981) [68] reported a severe attenuation of the circadian melatonin rhythm in adult rats exposed to ELF electric fields in the range of 2-40 kV/m for three weeks. The study measured the activity of pineal N-acetyltransferase and the melatonin content of the gland [68]. The subject was reviewed by Karasek and Lerchl (2002) [69]. Inversion of the GMF at night diminished the ability of the pineal gland to convert serotonin to melatonin. It is therefore plausible to speculate that diminished melatonin levels in SIDS could be related to the above-mentioned ELFs and could involve Schumann resonance given the correlations previously described by Cherry (2002) [51]. Alternatively, a metabolomic profiling study by Graham et al. [70] showed that the brains of SIDS cases contained novel predictive biomarkers, including ergothioneine, nicotinic acid, succinic acid, adenosine monophosphate, and azelaic acid. The latter is produced by bacterial degradation of nonanoic acid. Azelaic acid is a tyrosinase inhibitor and is known to inhibit melanin synthesis; thus, there is support for another mechanism (acting solely or in concert with ELFs) that is consistent with a bacterial infection in SIDS.

The broad biological [71], physiological, and health [72,73] effects of solar energy now make up a large part of the science of heliobiology [74]. It is now evident that solar energy interacts with human physiological processes, and this provides a novel putative contributor to SIDS causation.

Many human physiological processes are directly affected by solar energy emissions, including sunspots [51-53] and cosmic ray effects [54]. For instance, both systolic and diastolic blood pressures are affected [74]. Numerous other health-related events, including myocardial infarction [75], stroke, and sudden adult death, correlate strongly with sunspot activity. Evidence indicates that these conditions are related to an underlying inflammatory state [76].

The brain is an electromagnetic organ that receives protective and cellular repair support and anti-oxidative and anti-inflammatory properties of melatonin [77]. Solar/geomagnetic activity reduces melatonin and low levels correlate with both SSNs and increases in suicide, accidental death, and cerebrovascular (stroke) mortality [53]. Typically, low levels of melatonin in SIDS would likely decrease anti-inflammatory effects and increase the risk of infection and SIDS. This combination of infection/inflammatory state thus highlights the importance of the link between infection and the prone sleep position and the increased risk of SIDS [37-39] and could suggest an alternative mechanism in relation to sunspot/solar influences; however, further studies are needed to establish or refute a statistically significant relationship. Other mechanisms involving light could also play a role: the opsin family of G-protein-coupled receptors acts as light detectors in animals. Opsin 5 (neuropsin) is sensitive to visible violet light and is found in the retina and skin. It is expressed in the hypothalamic preoptic area and participates in brown fat thermoregulation [78].

Solar energy's effects on the gut microbiome remain unexplored, and it could offer possible links as certain gut bacteria are electrogenic [79]. Also, there has been new information in relation to sun exposure and changes in the gut microbiome [80,81]. As the gut microbiome plays an important role in immune system homeostasis [82], it could contribute to SIDS pathogenesis.

In exploring possible heliobiological effects, it is plausible that increased sunspot activity could selectively act in individuals who are in an inflammatory state and who could lack the protective effects of melatonin. Alternatively, the solar activity could influence the virulence of infecting agents, resulting in adverse outcomes in infected infants. Evidence suggesting that increased sunspot activity underlies epidemic and epizootic disease outbreak events [83,84] provides some support for the latter idea. The recent severe acute respiratory syndrome coronavirus 2 (SARS-CoV-2) pandemic hit at a time of low solar sunspot activity (solar minimum, but coincidentally, when the Earth’s magnetic field was weak and exposure to cosmic radiation was high); however, the evolution of the virus, which is the moment when it attained transmissibility from bats to humans, may have been assisted by sunspot activity and may have occurred when SSNs were high sometime in the past. The effect of coronavirus disease 2019 (COVID-19) on SIDS incidence is yet to be reported.

Despite decades of research, mainstream researchers have not been able to establish a connection between SIDS and reproducible and broad-sweeping neuropathological findings [40,41]. The only remarkably consistent gross pathological findings in SIDS are heavy fluid-laden lungs, intrathoracic petechial hemorrhages, and liquid (unclotted) blood, as described previously. These findings in ~90% of cases suggest a single pathological mechanism in all but 10% of cases: a situation that is in line with Occam’s razor. SIDS risk factors such as prone sleep position [85], vulnerable age and prematurity [33], male sex [86], sleeping on a mattress [87] or sofa [88] that has been used by others, co-sleeping [89], etc., all point to the possible involvement of infection. The obvious point is that the sleeping environment is more likely to cause infection through contact with bacterial or viral pathogens. The risk factor associated with prone sleep position, therefore, acts as a co-factor for infection and is not necessarily part of the lethal process, as claimed by mainstream researchers [36], since babies die of SIDS on their backs and on their sides. Many studies have confirmed strong links to specific bacterial and/or viral agents [89-96] in a significant proportion of SIDS cases, but these findings are not conclusive and have not been extensively described or correlated with gross pathological findings.

## Conclusions

Many decades of research have failed to provide a conclusive answer to the enigma of SIDS. This is unprecedented with respect to scientific endeavors in the 21st century. It is unfortunate that science has not found a cause(s) after years of active and intense investment in the SIDS question. This situation, by inference, suggests that a subtle influence could underlie the SIDS phenomenon. This review highlights previous, largely unrecognized publications on celestial effects and SIDS (and the largely ignored literature concerning infection and SIDS), which point to a plausible explanation that deserves serious investigation.

Given the evidence of the relationship between sunspots and deaths from various causes (sudden cardiac deaths, stroke, etc.) and the published findings on SIDS and sunspots, consideration should be given to possible common underlying solar-based phenomena. Further investigation and serious efforts are needed to devise new prevention strategies. Such efforts could initially focus on how solar electromagnetic energy influences the infected host and the infecting agent.
